# A New Take on John Maynard Smith's Concept of Protein Space for Understanding Molecular Evolution

**DOI:** 10.1371/journal.pcbi.1005046

**Published:** 2016-10-13

**Authors:** C. Brandon Ogbunugafor, Daniel L. Hartl

**Affiliations:** 1 Department of Organismic and Evolutionary Biology, Harvard University, Cambridge, Massachusetts, United States of America; 2 The Broad Institute of MIT and Harvard, Cambridge, Massachusetts, United States of America; Ontario Institute for Cancer Research, CANADA

## Abstract

Much of the public lacks a proper understanding of Darwinian evolution, a problem that can be addressed with new learning and teaching approaches to be implemented both inside the classroom and in less formal settings. Few analogies have been as successful in communicating the basics of molecular evolution as John Maynard Smith’s protein space analogy (1970), in which he compared protein evolution to the transition between the terms **WORD** and **GENE**, changing one letter at a time to yield a different, meaningful word (in his example, the preferred path was **WORD → WORE → GORE → GONE → GENE**). Using freely available computer science tools (Google Books *Ngram* Viewer), we offer an update to Maynard Smith’s analogy and explain how it might be developed into an exploratory and pedagogical device for understanding the basics of molecular evolution and, more specifically, the adaptive landscape concept. We explain how the device works through several examples and provide resources that might facilitate its use in multiple settings, ranging from public engagement activities to formal instruction in evolution, population genetics, and computational biology.

This is part of the *PLOS Computational Biology* Education collection.

## Background

Over 150 years since the publication of *On the Origin of Species*, evolutionary biology remains among the most influential ideas ever proposed, having transformed our understanding of the origin of life and the sources of biodiversity and having afforded new perspectives on sex [1,2], disease [3,4], and various aspects of social organization, cognition, and behavior [5,6]. More recently, breakthroughs in genomics and the rise of bioinformatics and computational biology have increased the reach of evolutionary thinking, as we can now ask questions at a finer level of detail than ever before. Despite these transformative modern lenses, the general public remains misinformed on basic aspects of molecular evolution, which reinforces scientific and technological knowledge gaps.

Innovative pedagogical tools, analogies, and thought experiments can greatly improve public understanding by reframing complicated scientific ideas into more familiar terms. Famous examples include the twins paradox (special relativity) [7], the Punnett square (genetics) [8], and Schrodinger’s cat (quantum mechanics) [9].

Evolutionary biology is a particularly challenging topic to teach, as its understanding requires population thinking [10] and statistical reasoning that can be difficult for the uninitiated (even those with a science background) to fully grasp. Even more, the ideological tides of the region where evolution is taught (e.g., a municipality’s stance on the teaching of evolution in schools and other related issues) can create a contentious (and sometimes uncomfortable) environment for everyone.

To improve how evolution is communicated to broader audiences, we need approaches based on intuitive analogies that can create bridges to complicated concepts in evolutionary biology. Specifically, we need tools to teach basic principles of molecular evolution, as this is the area in which many recent breakthroughs in evolutionary biology have taken place.

**Fig 1:**
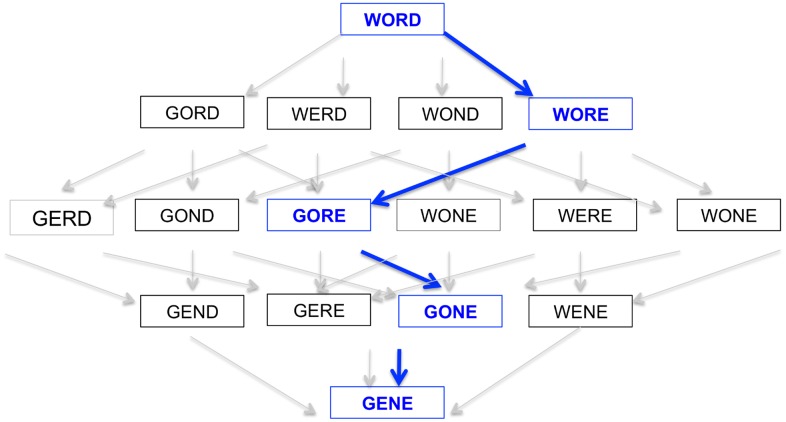
John Maynard Smith analogized evolution in protein space as a transition between WORD and GENE. This example elegantly illustrates how, despite an astronomical number of possible amino acid combinations, an emergent structure (encoded in protein space) exists that is sufficient for adaptive evolution by natural selection. Note that the above represents only a small subset of the entire protein space network. This subset contains 16 different “alleles,” for which only two different letters (W/G, O/E, R/N, D/E) are possible at each site. The actual alphabetical network space surrounding **WORD** is far greater: 4 individual sites, with 26 possible letters (of the English alphabet), creating a protein space with over 450,000 possible words (total size of the network = # of possible letters per site^total # of sites^ = 26^4^). In contrast, the limited network depicted in the figure has 2^4^ = 16.

The protein space analogy of John Maynard Smith (JMS) provides a creative and entertaining approach to introducing the fundamentals of molecular evolution. In this analogy, he compared protein evolution to a word game, in which the goal is to transform one meaningful word into another (**WORD** into **GENE)** by sequentially changing single letters at a time (Fig 1) [11]. He published this as a rebuttal to an argument suggesting that undirected natural selection is an inadequate sorting mechanism for the evolution of highly specialized, functionally adapted protein molecules [12]. Maynard Smith’s answer was that functional molecules are not located at random but rather are connected within a network (like the words in the game), which makes moving between different functional variants more feasible. In this scenario, variation in protein function arises via mutation. Should a variant exist in an environment in which it is more fit than a mutational neighbor, that variant will be represented in future generations in higher proportion. Through this incremental, algorithmic process, evolution by natural selection can create diverse proteins functionally equipped to solve a breadth of environmental challenges.

So powerful is Maynard Smith’s concept of protein space that it might be described as the substrate for other central ideas in evolutionary biology. Chief among these is Sewall Wright’s famed adaptive landscape [13], a conceptual and visual interpretation of the relationship between genotypes and phenotypes through which we can better understand microevolution. In the adaptive landscape, alleles corresponding to measurable values of some trait (phenotype) are connected via mutation, not unlike the words in Maynard Smith’s protein space. Wright’s adaptive landscape concept was one of the signature breakthroughs of the modern evolutionary synthesis [14] and has been put into experimental practice in modern studies of empirical adaptive landscapes [15–22].

Here, we revisit JMS’s protein space analogy, recast it in terms of the adaptive landscape, and intersect this hybrid analogy with values generated from the Google Books *Ngram* Viewer [23] to introduce an approach for exploring foundational principles in molecular evolution. We will use it to reconstruct hypothetical adaptive landscapes for several word transitions across a range of time environments. In doing so, we highlight how our updated analogy embodies several cutting-edge topics in evolutionary biology, including genotype by environment (G × E) [24] and adaptive landscape by environment interactions [21]. In addition to discussing the basics of the approach, we provide several entry points for communicating or teaching this material at various levels (see Supporting Information).

## An Updated JMS Analogy for Introducing the Adaptive Landscape

Although the original JMS analogy was successful in conveying the feasibility of evolution through sequence space, it did not focus on any particular case of adaptation between an ancestor and a fitness optimum, nor did it address why some trajectories through sequence space are preferred to others (questions that are central to the adaptive landscape). To fully hybridize the protein space analogy with the adaptive landscape, the individual alleles in our network (**WORD**, **WORE**, **GORE, GONE**, **GENE**) should be assigned a quantitative value to serve as a fitness proxy. With fitness values assigned, evolution from **WORD** to **GENE** (or any other hypothetical transition) would be feasible only if there was an increase in fitness at each step in the **WORD → GENE** transition. Even more, one could determine if, and possibly by how much, some pathways were preferred to others.

### Google Books *Ngram* Viewer

For proxy fitness values in our model, we utilize data from the Google Books *Ngram* Viewer, a tool inspired by an older prototype, called *Bookworm*, developed by researchers from Harvard University’s Cultural Observatory [25,26]. In this venture, over 15 million books have been digitized (and growing), 5 million of which were chosen for computational analysis, with word usage frequencies computed between 1800 and 2000. Single-word usage frequencies are calculated as the number of appearances of a word in a year divided by the total number of words in the entire analyzed word set (corpus) that year (see Box 1) [26]. Before further explaining the updated JMS analogy, we’ll define some basic terms and concepts from evolutionary biology and computer science that we’ll use for the remainder of this manuscript:

**Box 1. What is the Google Books *Ngram* Viewer?**

The Google Books *Ngram* Viewer allows one to trace the usage of individual words, or *n-grams*, though time for a number of languages. It was inspired by a prototype, called *Bookworm*, invented by scientists at the Harvard Cultural Observatory and the Massachusetts Institute of Technology. It is a valuable tool in computational linguistics, digital humanities, and “culturomics,” all relatively new fields that use computer science and advanced algorithms to study questions historically relegated to the humanities and social sciences. In 2013, the inventors of *Bookworm* published a popular book (*Uncharted*: *Big Data as a Lens on Human Culture*, Penguin Books, 2013) that describes the origins of the idea and takes readers through several examples of questions that can be addressed with the Google Books *Ngram* viewer (https://books.google.com/ngrams). The central metric in the viewer is the *n-gram* frequency score: the frequency of a word’s appearance in a given year.

$$n-gram\text{ frequency score}=\;\frac{\text{Total \# of times a given word (}n-gram\text{) appeared in all books in a year }}{\text{Total \# of words in all analyzed books (corpus) in that year }}$$

The *Google Books Ngram* viewer gives users *n-gram* frequency scores in the form of a graph, with the *n-gram* frequency score on the *y*-axis, and the year on the *x*-axis. In this study, we use the *n-gram* frequencies to generate “fitness” values for words in our hypothetical word adaptive landscapes. The scores are analogized as “*n-gram* fitness” in our model but are the same as the raw *n-gram* frequency scores. (For an example, see S1A Fig in S1 File.)

### Adaptive landscape

Throughout this study, we use the term “adaptive landscape” rather than the related “fitness landscape.” Scientists might prefer one of these terms for their own reasons, but they are essentially the same and interchangeable.

### Accessible trajectory

The term “accessible trajectory” refers to a path across an adaptive landscape towards a fitness peak where each successive allele has a higher fitness than the one preceding it (as in, fitness is increasing along a path). Inaccessible trajectories are those interrupted by a steep “fitness valley,” which can constrain evolution along certain pathways.

### N-gram

Most simplistically, an *n-gram* can be described as a continuous sequence of letters uninterrupted by a space. In the JMS analogy, the individual words (**WORD**, **WORE**, etc.) are 1-grams (corresponding to one uninterrupted sequence) and are likened to a sequence of amino acids in a specific protein variant (an allele). This could also apply to a continuous chain of nucleotides that make up a DNA or RNA molecule.

### Fitness proxy

The case-insensitive frequency score for a word, or *n-gram* score, is the quantity that will be used as the fitness value for a particular allele in our updated analogy. Note that the units in the *n-gram* viewer are in terms of frequency of word use but are analogized as reproductive fitness in the teaching exercise. The frequency score is solely and exclusively a means to generate numbers for words to add depth to the original JMS analogy by transforming it into an adaptive landscape. We find *n-gram* frequency scores to be an appropriate fitness proxy for the words in our hypothetical adaptive landscapes because they assign unambiguous values to words according to a clear and transparent method, and these values are accessible to almost anyone. It also lends itself to gamification and entertaining exercises that can be used in various settings (see Supporting Information).

### Environment

We analogize a word’s change in the *n-gram* frequency score from the Google Books *Ngram* Viewer as changes in fitness of an allele as a function of the environment (time = environment). This allows one to study genotype by environment interactions [24] with this device.

## The Updated Analogy in Practice: Two Examples

### Example I: The original WORD → GENE transition as an adaptive landscape

**Fig 2:**
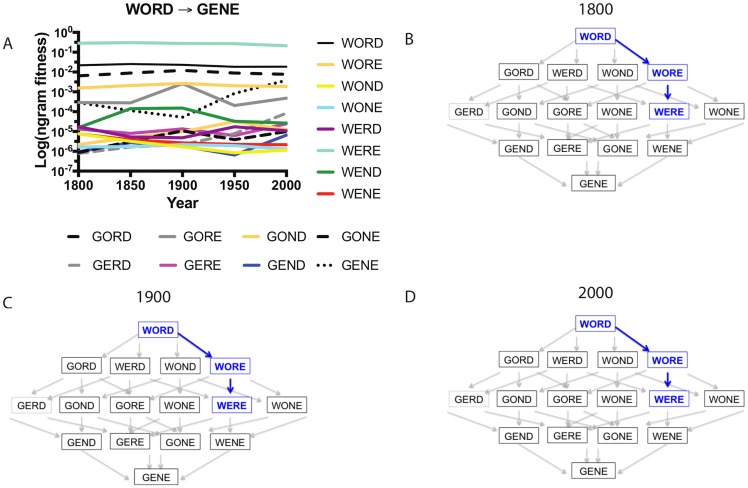
The accessibility of adaptive trajectories: using the updated analogy, the WORD → GENE transition becomes trapped at the WERE intermediate across environments. (A) *n*-*gram* fitness values for the alleles composing the landscape across environments (years). Note that the values on the *y*-axis are plotted on a log scale, and so the relationship between words will appear different than their representation when graphed using the Google Books *Ngram* Viewer online tool (for which the *y*-axis is not on a log scale). Trajectory figures for (B) 1800, (C) 1900, and (D) 2000 summarize the results of simulations of evolution across that landscape. By exploring the **WORD → GENE** transition as an adaptive landscape, we learn that evolution takes an alternate route, from **WORD → WERE**, and remains trapped on that peak across all environments (years). The **WORE** “intermediate” never reaches fixation but is a stepping stone allele through which the high fitness **WERE** allele arises. Computer simulations of this evolution are discussed in the Supporting Information (S3A–C Fig in S3 File).

Using *n-gram* frequency values as a proxy for fitness, let us examine the adaptive landscape for transitions between **WORD** and **GENE** (as in Fig 1). We should note that four-letter landscapes of this kind are similar to several experimental systems and, in particular, studies of antimicrobial resistance (see Box 2). In these four-letter landscapes, there are 4! = 24 possible pathways between the first (**WORD)** and last term (**GENE)** across the adaptive landscape. Fig 2 depicts the overall inaccessibility of pathways from **WORD** to **GENE** across environments (years). This is because of the intermediate **WERE**, which has a much higher *n-gram* fitness (orders of magnitude) than any word in the entire landscape (Fig 2, S2A Table in S2 File). This suggests that an evolutionary process that attempts to move from **WORD** to **GENE** using accessible trajectories would likely get trapped on the **WERE** peak at all years between 1800 and 2000. Note that in this example, **WORD** has higher *n-gram* fitness than the intermediate **WORE**, which might render any pathway leaving **WORD** inaccessible. Evolution at high mutation rates, however, would generate enough **WORE** intermediates (in low frequency) that the mutant neighbor **WERE** could be located eventually. In this scenario, the **WORE** intermediate would never appear in high abundance, and a simulation of the entire landscape evolving over generation time would show a population initially dominated by **WORD** transitioning to **WERE** almost instantly (S3A–C Fig in S3 File). In this example, we observe a phenomenon akin to “stochastic tunneling,” in which intermediate steps in trajectories appear to be skipped over during evolution at high mutation rates and large population sizes [27,28]. Of course, the **WORE** intermediate is not skipped over, but only appears long enough for **WERE** to arise by mutation, which then quickly overtakes the population (before **WORE** has an opportunity to rise to an appreciable frequency).

**Box 2. The Adaptive Landscape: Applications to the Evolution of Drug Resistance**

The adaptive landscape concept has been put to practical use in several ways, most notably towards understanding the evolution of antimicrobial drug resistance. In a landmark study, Daniel M. Weinreich and colleagues (2006) identified the most likely pathways towards the evolution of maximal drug resistance in bacteria [15] by using an approach similar to the hybrid protein space/adaptive landscape JMS analogy being proposed here. They created a collection mutants of a resistance protein corresponding to all possible mutation combinations connecting a wild-type, drug-susceptible variant (analogous to **WORD** in the JMS example) to the most drug-resistant variant of that protein (analogous to **GENE**), with all mutants possessing an experimentally determined value for a fitness proxy (in this case, a measure of how well that protein variant performs in the presence of a very high dose of antibiotic drug). Computer simulations helped to identify the most likely pathways between the susceptible and resistant protein variants. This study inspired a series of follow-ups that used a similar approach to identify probable pathways in the evolution of drug resistance in other microbes. One study applied this method to a model system for malarial resistance to the drug pyrimethamine (see S1B Fig in S1 File) [16].

This example illustrates how the introduction of a simple fitness proxy makes a clear distinction between what mutation does (it changes a letter) and what natural selection does (it allows or disallows a change according to whether it increases or decreases fitness). With the *n-gram* fitness proxy, the transition from **WORD** to **GENE** is no longer possible, because there are no paths of stepwise increase in fitness between **WORD** and **GENE**. Instead, evolution at high mutation rates and/or large population sizes would favor a single trajectory (**WORD → WORE → WERE)** with a small likelihood that random processes (genetic drift) would drive a population from the **WERE** fitness peak to lower fitness parts of the landscape.

### Example II: GENE → BIRD transition as an adaptive landscape

Having examined the original **WORD → GENE** example to discuss the basic concepts of mutation and selection, we can now highlight other properties of adaptive landscapes. To do this, we’ll use an example that offers new considerations: the transition from **GENE** to **BIRD**.

**Fig 3:**
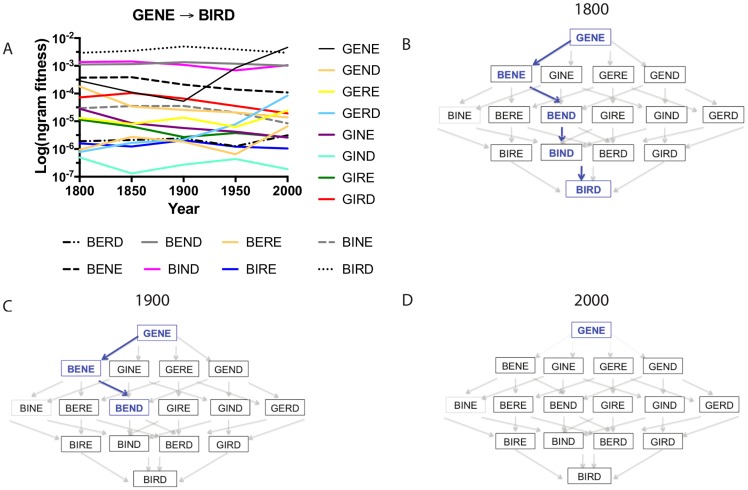
Adaptive landscape by environment interactions: using the updated analogy, we can observe how the structure of adaptive landscapes changes as a function of environment for the transition GENE → BIRD. (A) *N-gram* fitness values for the alleles composing the landscape across environments (years). This demonstrates how the structure of the adaptive landscape topography changes as a function of environment (year). As in Fig 2, the values on the *y*-axis are plotted on a log scale. Trajectory figures correspond to (B) 1800, (C) 1900, and (D) 2000. In 1800, there is an accessible pathway from **GENE** to **BIRD**. By 2000, **GENE** has such high *n-gram* fitness that a population fixed for individuals of the **GENE** “allele” might remain trapped on a fitness peak, unable to move via selection to other nodes on the landscape. Computer simulations of this evolution are discussed in the Supporting Information (S3D–F Fig in S3 File).

As in the prior example, each of the four letters must be changed one letter at a time. Hence, in any transition from **GENE** to **BIRD**, the **G** changes to **B**, the first **E** to **I**, the **N** to **R**, and the second **E** to **D**. The total number of 4-letter words with either **G** or **B** in the first position, **E** or **I** in the second, **N** or **R** in the third, and **E** or **G** in the fourth is 2 (choices per site) raised to the power of 4 (letters in the word), or 16. Using the *n-gram* frequency scores, we can construct a graph and table with the *n-gram* fitness values for the individual terms of a word landscape in differing environments corresponding to *n-gram* values for different years between 1800 and 2000 (Fig 3, S2B Table in S2 File).

In Fig 3, we observe that in the **GENE** → **BIRD** transition, the pathway (**GENE** → **BENE** → **BEND** → **BIND** → **BIRD**) shows increasing fitness at each step for the year 1800, qualifying as an accessible trajectory. This pathway is defined by one notable peculiarity: the word **BENE** (Latin for “well”) is rarely used in English texts today but, prior to 1928, was used more frequently than **GENE** [23]. This is because the word **GENE** (as defined in biology) was first coined in 1909 [29] and rapidly increased in usage along with the growth of genetics as a scientific field. Because of changing word usage, by 1950, the stepwise evolution from **GENE** to **BIRD** would be improbable, because the starting point, **GENE**, had higher *n-gram* fitness than any of its single mutant neighbors (**BENE**, **GINE**, **GERE**, or **GEND**) (S2B Table in S2 File). So drastically does the **GENE** to **BIRD** landscape change that, by 2000, **GENE** has higher *n-gram* fitness than **BIRD** (Fig 3), itself a common English word (and revealing of a striking cultural shift: today we write about the “gene” more than we do the “bird”). We would describe the changing topography of the landscape as having environmental dependence or as demonstrating “an adaptive landscape by environment interaction,” a product of the collective gene by environment interactions for the alleles composing the adaptive landscape [21].

## Summary

We have used a freely available platform to enrich a venerable change-one-letter-game analogy for protein evolution invented by JMS. By adding quantitative fitness values to the “alleles” in word transition adaptive landscapes, we can transform any number of word transition problems to an apt model for the process of molecular evolution.

Through these examples, we hope to empower a new generation of students, citizens, and scientists to develop an intuitive appreciation for the process of evolution. We want to emphasize that the updated analogy is designed to provide insight into the principles of biological evolution and has no relevance to the field of evolutionary linguistics, which operates on different principles [30,31]. And, although we should use caution when applying the analogy, even in other biological contexts, the device can easily be modified to address more advanced topics in evolutionary biology (see Box 3). For example, it would be simple to use *n*-*gram* fitness values to calculate the sign and magnitude of epistasis based on the combined effects of multiple letter substitutions in certain words (analogous to genetic backgrounds) [32,33] or to explore concepts such as landscape ruggedness [34] and network principles like robustness and evolvability [35].

**Box 3. Concepts at a Glance**

The approach might be particularly attractive for teachers because it can be used to explain basic principles of molecular evolution to students at various stages. We’ll divide the concepts into three classes—beginner, intermediate, and advanced.

**Beginner:** Students at this level should only be using the simplest version of the approach. This is most appropriate for citizen-scientists, secondary school, and college biology/evolution courses designed for non-scientists.

- The central dogma of biology
- Basic molecular biology
- The basics of Darwinian evolution

**Intermediate:** Students at this level should have been exposed to college-level biology

- Intermediate molecular biology
- Basic population genetics
- The adaptive landscape

**Advanced:** Students are upper-division–level undergraduates or graduate students in biology or evolution

- Advanced population genetics
- Epistasis
- Computational biology (modeling evolution)

As the original JMS analogy didn’t require an advanced mathematical or computer science background to comprehend, this update to the analogy requires only a personal computer with a browser and Internet access (see Box 4). This is a key feature of the tool, as it hopes to bridge existing gaps in technology, coding experience, and computer science familiarity that can serve as barriers to entry in the computational sciences. More advanced technology might be useful for automating the operations outlined above, but possibly at the expense of the insight that is gained from hands-on experimentation. However, in the Supporting Information, we provide illustrative examples of how existing open-source computational biology tools can be used to model several of the examples used in the main text. Interested readers can follow additional work and progress on this device through resources provided in the Supporting Information.

**Box 4. Teaching and Exploring Tool Box**

Use of this updated analogy as a teaching or exploration device requires only basic computing skills and technology.

**For beginner usage**:

- Personal computer
- Access to Google Books *Ngram* Viewer

**For intermediate usage**:

- Basic graphing and statistics program to visualize pathways and calculate basic properties like mean fitness of an “allele”

**For advanced usage**:

- Access to more advanced computer simulation packages
- Proficiency in programming languages like python, R, MATLAB, Mathematica, and countless others

This manuscript is written so that readers can understand the updated JMS protein space analogy and use it to explore, teach, and learn fundamentals of molecular evolution. Additional data, discussion, and resources are included in the Supporting Information materials, which we encourage interested readers to explore. They include the following:

- Figures corresponding to the contents of Boxes 1 and 2
- Data tables for the examples used in the main text
- Illustrative examples of computer simulations of evolution in the word adaptive landscapes discussed in the main text
- Several teaching-focused materials, including the following:
  - Two teaching exercises that can be used in the classroom
  - A presentation that explains the tool, aimed towards anyone interested in using it to teach evolution at various levels
- A list of additional publications exploring adaptive landscapes across a broad number of scientific contexts
- A supplementary two-letter *n-gram* example to further illustrate how this device can be applied to an even simpler situation

A longer-term goal is to make this project an ongoing and collaborative one in which the scientific community can exchange new perspectives, applications, evaluation tools, and findings. Readers can follow progress on this project at scholar.harvard.edu/chike98 and on Twitter: @Word2Gene. Interested readers are urged to contact the authors with questions and are welcome to create dialogue around the tool in open science spaces and on social media.

## Supporting Information

S1 File Figures corresponding to the information in Boxes 1 and 2. (DOCX)

S2 File Data tables for word transition landscapes as discussed in the main text. (DOCX)

S3 File Computational Biology: simulations using simuPOP. (DOCX)

S4 File Teaching exercises. (DOCX)

S5 File Slideshow presentation introducing the analogy and learning device. (PDF)

S6 File Additional references on adaptive landscapes. (DOCX)

S7 File Two-letter abbreviations for US states example. (DOCX)
