# Supplementary material for: A New Take on John Maynard Smith's Concept of Protein Space for Understanding Molecular Evolution
Source: PLoS Comput Biol. 2016 Oct 13;12(10):e1005046. doi: 10.1371/journal.pcbi.1005046 (PMC5063322; doi:10.1371/journal.pcbi.1005046)
Supplement: S1 File — (DOCX) [file pcbi.1005046.s001.docx]

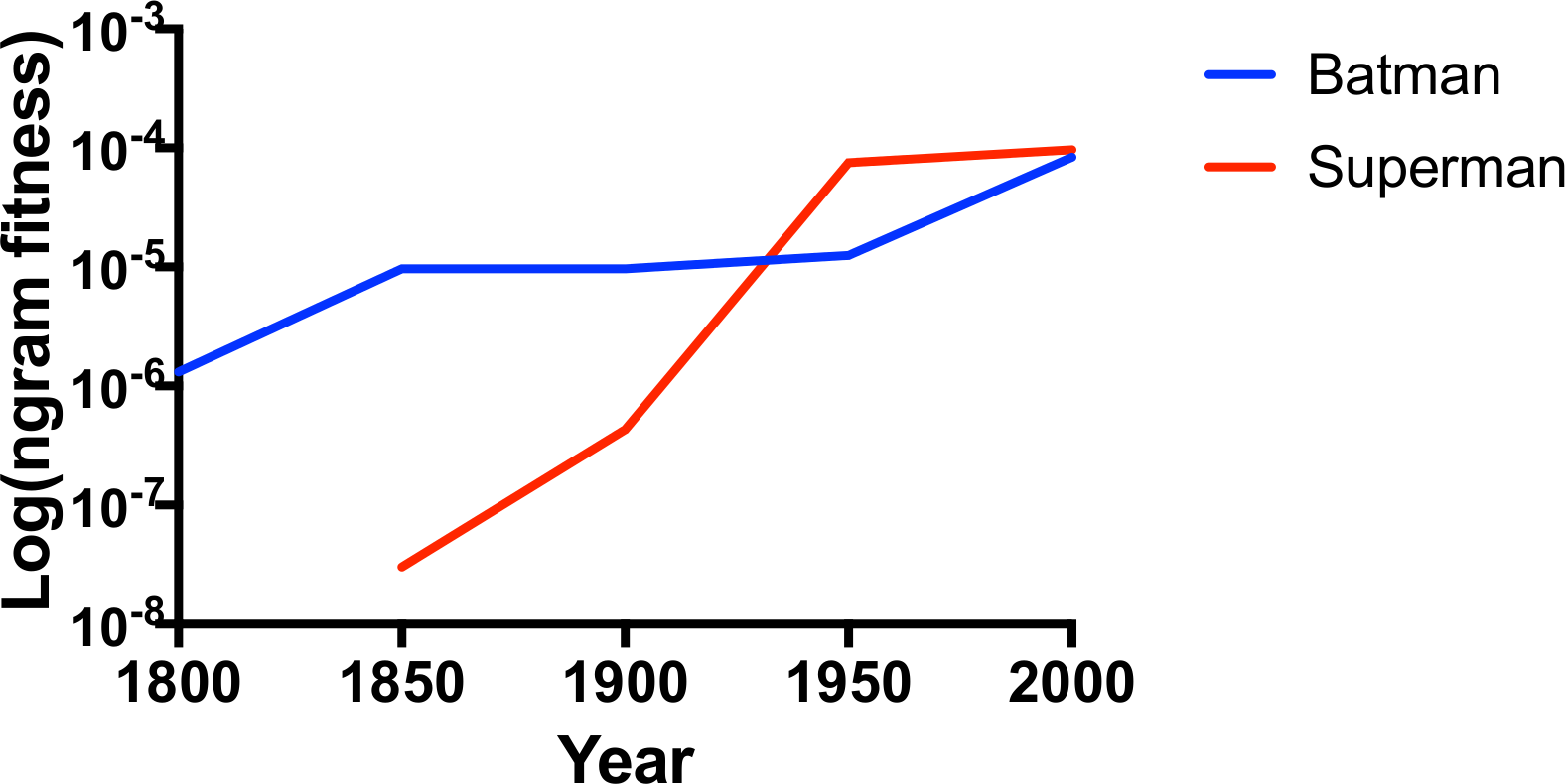


**S1A Fig. An example of the data generated by the Google Books *Ngram* Viewer. This corresponds to Box 1.** Here we observe changes in the usage of the words “Batman” and “Superman” in English through time (case-sensitive). A “culturomic” perspective might use these data to study cultural trends pertaining to these two superheroes. For example, one could ask how “Batman” was used prior to the creation of the popular comic book character of the same name, and how that event changed the word’s usage. In this study, we analogize the frequency score as “*n-gram* fitness” as observed on the *y*-axis. Note that the values on the *y*-axis are plotted on a log scale, and so the trends in this graph appear different than the graphs returned from the Google Books *Ngram* Viewer online tool.

**
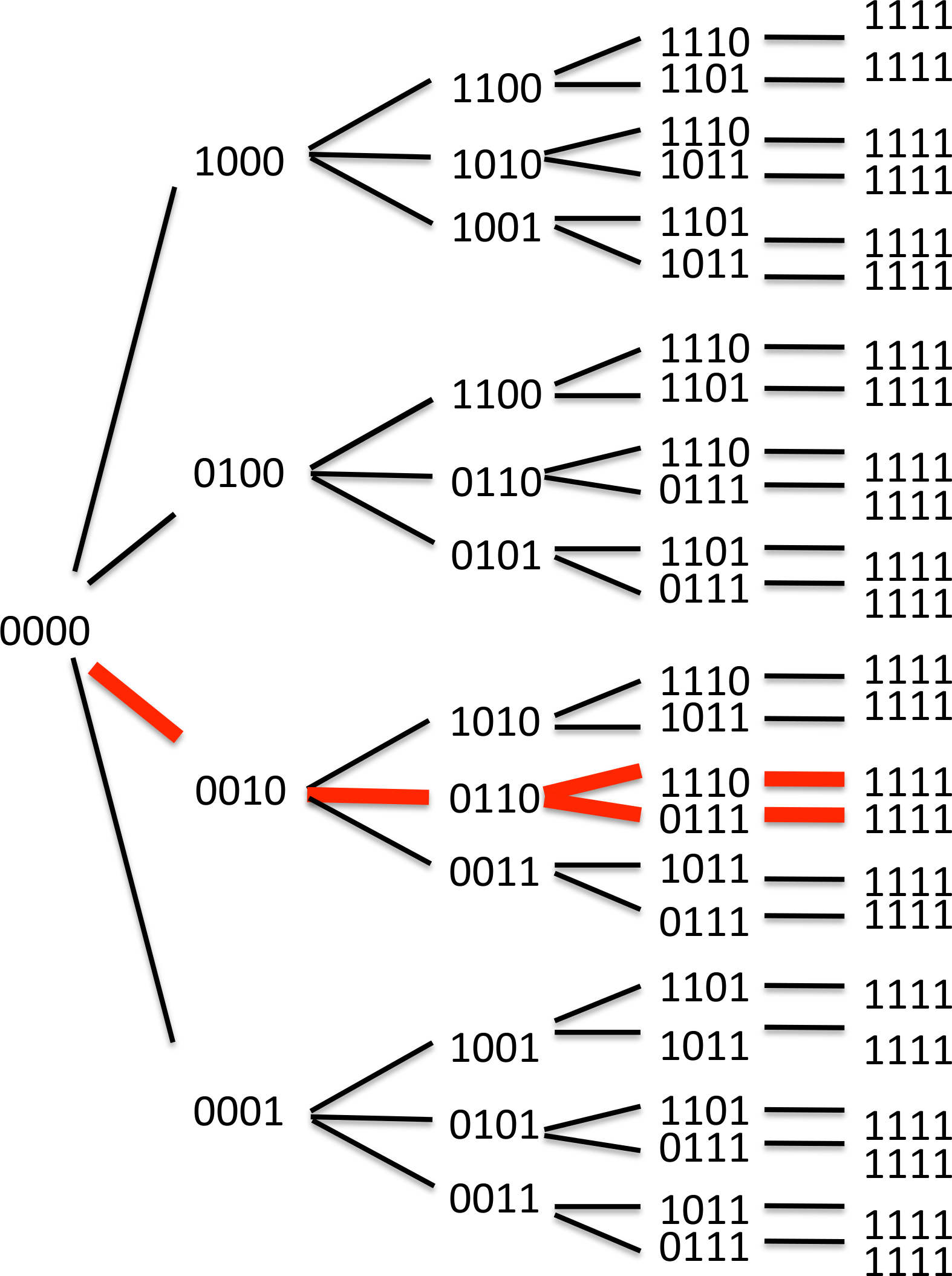
**

**S1B Fig. Summary of a main result from Lozovsky et al. 2009. This corresponds to Box 2.**  Starting from the wild type (0000) genotype, researchers identified the preferred pathways towards maximal drug resistance (1111) for a protein determinant of drug resistance (dihydrofolate reductase, an enzyme) in *Plasmodium falciparum*. For more, refer to: Lozovsky ER, et al. Stepwise acquisition of pyrimethamine resistance in the malaria parasite. Proceedings of the National Academy of Sciences of the United States of America. 2009 Jul 21;106(29):12025–30)
