# Supplementary material for: A New Take on John Maynard Smith's Concept of Protein Space for Understanding Molecular Evolution
Source: PLoS Comput Biol. 2016 Oct 13;12(10):e1005046. doi: 10.1371/journal.pcbi.1005046 (PMC5063322; doi:10.1371/journal.pcbi.1005046)
Supplement: S2 File — (DOCX) [file pcbi.1005046.s002.docx]

**Supplemental file 2**

C. Brandon Ogbunugafor and Daniel L. Hartl

***A New Take on John Maynard Smith's Concept of Protein-Space for Understanding Molecular Evolution***

**Data tables for word transition landscapes as discussed in the main text**

In this section, we provide the *n-gram* fitness values for the words used to construct the landscapes as described in the main text.

| **Binary** | **Four-letter 1-Gram**  **(“Allele”)** | **1-gram frequency score**  **(Year 1800)** | **1-gram frequency core**  **(Year 1850)** | **1-gram frequency score**  **(Year 1900)** | **1-gram frequency score**  **(Year 1950)** | **1-gram frequency score**  **(Year 2000)** |
| --- | --- | --- | --- | --- | --- | --- |
| 0000 | WORD | 2.20E-02 | 2.55E-02 | 2.31E-02 | 1.85E-02 | 1.87E-02 |
| 0001 | WORE | 1.55E-03 | 2.09E-03 | 2.66E-03 | 2.02E-03 | 1.87E-03 |
| 0010 | WOND | 7.68E-06 | 3.19E-06 | 1.70E-06 | 8.42E-07 | 1.14E-06 |
| 0011 | WONE | 1.65E-06 | 1.75E-06 | 2.19E-06 | 1.92E-06 | 1.31E-06 |
| 0100 | WERD | 1.73E-05 | 5.30E-06 | 4.79E-06 | 1.72E-05 | 1.09E-05 |
| 0101 | WERE | 2.85E-01 | 3.04E-01 | 2.78E-01 | 2.69E-01 | 2.11E-01 |
| 0110 | WEND | 1.58E-05 | 1.43E-04 | 1.51E-04 | 3.30E-05 | 2.64E-05 |
| 0111 | WENE | 7.58E-06 | 3.77E-06 | 2.68E-06 | 2.25E-06 | 2.16E-06 |
| 1000 | GORD | 8.76E-07 | 3.19E-06 | 1.08E-05 | 3.90E-06 | 1.00E-05 |
| 1001 | GORE | 2.90E-04 | 2.80E-04 | 2.57E-03 | 2.05E-04 | 4.84E-04 |
| 1010 | GOND | 2.22E-06 | 4.87E-06 | 9.69E-06 | 3.17E-05 | 1.23E-05 |
| 1011 | GONE | 6.41E-03 | 8.91E-03 | 1.22E-02 | 8.90E-03 | 7.71E-03 |
| 1100 | GERD | 7.83E-07 | 1.67E-06 | 2.19E-06 | 7.85E-06 | 8.47E-05 |
| 1101 | GERE | 1.31E-05 | 7.98E-06 | 1.36E-05 | 6.24E-06 | 2.44E-05 |
| 1110 | GEND | 9.46E-07 | 2.74E-06 | 1.82E-06 | 6.48E-07 | 6.59E-06 |
| 1111 | GENE | 2.88E-04 | 1.13E-04 | 5.29E-05 | 8.37E-04 | 3.84E-03 |

**S2A Table.** *N-gram* fitness values for the individual alleles in the WORD🡪GENE transition landscape.

| **Binary** | **Four-letter 1-Gram**  **(“Allele”)** | **1-gram frequency score**  **(Year 1800)** | **1-gram frequency core**  **(Year 1850)** | **1-gram frequency score**  **(Year 1900)** | **1-gram frequency score**  **(Year 1950)** | **1-gram frequency score**  **(Year 2000)** |
| --- | --- | --- | --- | --- | --- | --- |
| 0000 | GENE | 2.88E-04 | 1.11E-04 | 5.29E-05 | 8.37E-04 | 4.63E-03 |
| 0001 | GEND | 9.46E-07 | 2.74E-06 | 1.82E-06 | 6.48E-07 | 6.59E-06 |
| 0010 | GERE | 1.31E-05 | 8.00E-06 | 1.36E-05 | 6.24E-06 | 2.44E-05 |
| 0011 | GERD | 7.83E-07 | 1.67E-06 | 2.19E-06 | 7.85E-06 | 8.47E-05 |
| 0100 | GINE | 2.83E-05 | 8.51E-06 | 5.70E-06 | 4.19E-06 | 2.59E-06 |
| 0101 | GIND | 4.94E-07 | 1.31E-07 | 2.74E-07 | 4.37E-07 | 1.88E-07 |
| 0110 | GIRE | 1.14E-05 | 6.49E-06 | 2.70E-06 | 3.78E-06 | 2.64E-06 |
| 0111 | GIRD | 7.25E-05 | 1.05E-04 | 6.61E-05 | 3.55E-05 | 1.92E-05 |
| 1000 | BENE | 3.75E-04 | 3.89E-04 | 2.10E-04 | 1.39E-04 | 1.10E-04 |
| 1001 | BEND | 1.12E-03 | 1.14E-03 | 1.35E-03 | 1.19E-03 | 1.01E-03 |
| 1010 | BERE | 1.81E-04 | 3.39E-05 | 2.58E-05 | 2.09E-05 | 1.38E-05 |
| 1011 | BERD | 1.91E-06 | 2.12E-06 | 2.41E-06 | 1.27E-06 | 3.12E-06 |
| 1100 | BINE | 2.98E-05 | 3.54E-05 | 3.55E-05 | 2.08E-05 | 8.48E-06 |
| 1101 | BIND | 1.37E-03 | 1.44E-03 | 1.09E-03 | 6.87E-04 | 1.06E-03 |
| 1110 | BIRE | 1.59E-06 | 1.23E-06 | 2.07E-06 | 1.22E-06 | 1.04E-06 |
| 1111 | BIRD | 2.96E-03 | 3.47E-03 | 4.97E-03 | 3.94E-03 | 2.95E-03 |

**S2B Table.** *N-gram* fitness values for the individual alleles in the GENE🡪BIRD transition landscape. Note that these are case insensitive, because unlike the state abbreviations, there is no standard form type for these words in their common usage. Note how GENE has lower usage than BENE for several dates. In the actual Google Books *Ngram* Viewer, GENE only overtakes BENE in usage in 1928. As of 1927, the *n-gram* frequency for BENE was 2.459E-04 and 2.451E-4 for GENE. As outlined in the main text, this change alters the accessibility of trajectories across the landscape, indicative of an “adaptive landscape by environment interaction.”
