# Supplementary material for: A New Take on John Maynard Smith's Concept of Protein Space for Understanding Molecular Evolution
Source: PLoS Comput Biol. 2016 Oct 13;12(10):e1005046. doi: 10.1371/journal.pcbi.1005046 (PMC5063322; doi:10.1371/journal.pcbi.1005046)
Supplement: S3 File — (DOCX) [file pcbi.1005046.s003.docx]

**Supplemental file 3**

C. Brandon Ogbunugafor and Daniel L. Hartl

***A New Take on John Maynard Smith's Concept of Protein-Space for Understanding Molecular Evolution***

**Computational Biology: Simulations using simuPOP**

Though we aimed for this manuscript (and the lessons it contains) to be interpretable by anyone with a basic knowledge of biology, we wanted to highlight existing freely available computational tools such that those interested could expand on the content for their own exploratory or educational purposes. For example, these simulations could be built into the basic lessons introduced in the main text, and even serve as a way to bridge the teaching of molecular evolution with an introduction to computational biology.

Here we discuss the results of simulations of evolution across the landscapes discussed in Figs. 2 and 3 (main text). In doing so, we explore another way to use the JMS analogy to study evolution: through computational tools that have been used in studies of empirical adaptive landscapes. This exercise is not intended as a rigorous examination, but more of a simple demonstration of the kinds of things that one can do with existing tools.

There are a number of different methods that one could use to simulate evolution, written using different computing languages and simulation platforms. Many experienced computer scientists who study evolution often write their own code. Here we’ll introduce a package called simuPOP [1], which is freely available and has excellent online resources that one can consult for help. It has been used to simulate evolution in several scenarios [2–5] and is very flexible: aspects of it (population size, mutation rate, length of simulation, many others) can be tweaked to fit the system that one is interested in simulating.

We suggest that those interested in simuPOP should consult the extensive online resources available. Taking full advantage of the package requires a working understanding of Python, one of the computer languages that one can use to create these kinds of tools. There are hundreds of online courses and resources available for learning Python. We urge you to explore some of the more popular ones: Google’s Python Class, Udacity, Coursera, and other resources that can easily be found on the Internet.

S3A-F Figs. below highlight the results of simulations in simuPOP, under a set of conditions specific to a given evolutionary scenario: length of simulation, population size, and mutation rate. These details are highlighted in the figure legends.

**
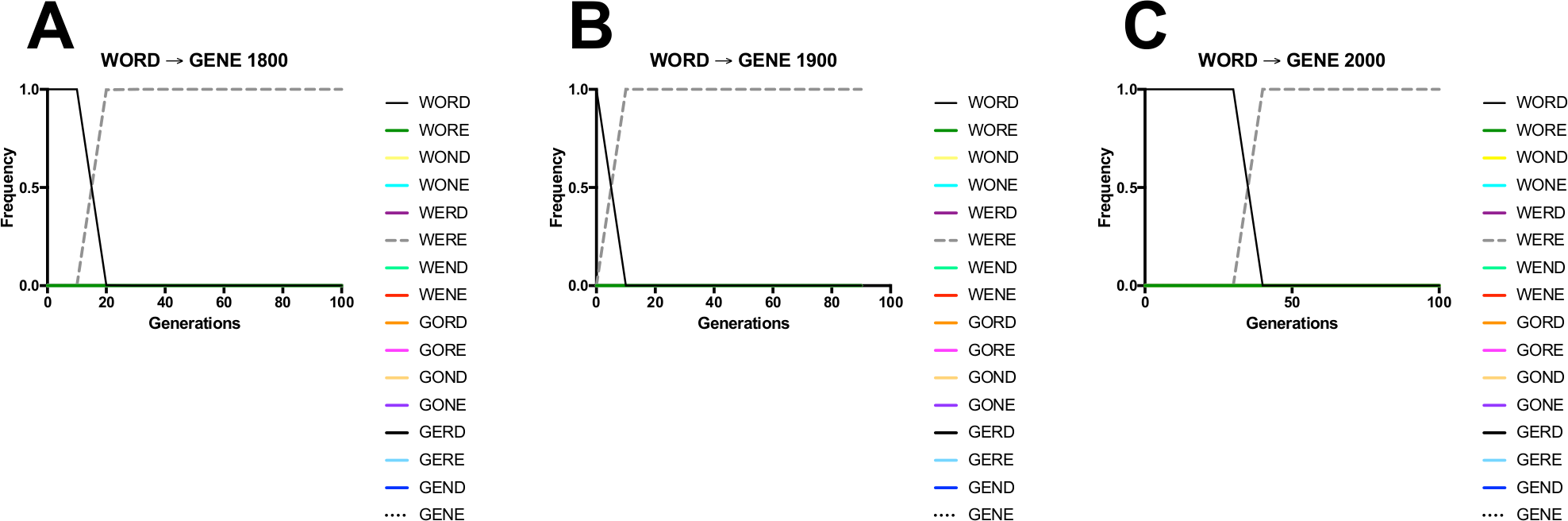
**

**S3A-C Figs.** **Simulations of evolution across the WORD🡪GENE landscape.** These are illustrative examples of the most preferred pathways for evolution at each of the simulated environments. Panels correspond to several simulation scenarios: (A) 1800, (B) 1900 (C) 2000. The higher mutation rate allows the population to search a broader segment of sequence space, and so even alleles that are of lower fitness appear relatively frequently. This is important in this evolutionary scenario because the “stepping stone” allele in this process, **WORE**, exists in such low frequency that it doesn’t appear in these frequency plots at all. It is generated from **WORD**, and generates mutants of its own, one of which is the **WERE** fitness peak. After being generated, **WERE** almost instantly takes over the population. From one vantage point (simply looking at frequency space as depicted in the graphs), it appears like evolution skipped from **WORD** directly to **WERE**, 2 mutations away. In fact, **WORE** wasn’t skipped, but rather, appears in very low frequency before locating the **WERE** fitness peak.


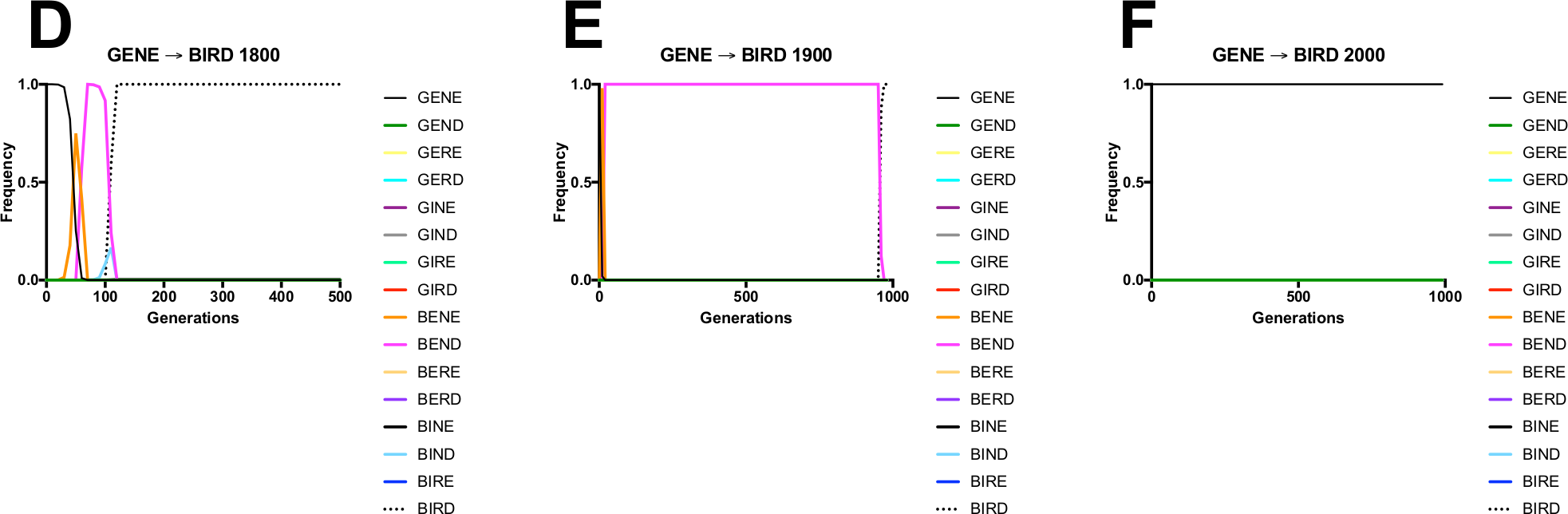


**S3D-F Figs.** **Simulations of evolution across the GENE🡪BIRD landscape.** These are illustrative examples of simulations of evolution across 3 environments: (D) 1800 (E) 1900 (F) 2000. The results of simulations are representative of dynamics discussed in the Fig. 3 (main text), with full traversal of the landscape in 1800 (**GENE🡪BENE🡪BEND🡪BIND🡪BIRD**). In 1900, the landscape eventually reaches the **BIRD** peak but only after 900 generations of evolution, much of it trapped on the **BEND** local peak. In 2000, however, the population is trapped, from the outset and indefinitely, on the **GENE** peak. This is because, in 2000, **GENE** has the highest *n-gram* fitness of any word in the entire landscape (see S2B Table).

**References**

1. Peng B, Amos CI. Forward-time simulations of non-random mating populations using simuPOP. Bioinforma Oxf Engl. 2008 Jun 1;24(11):1408–9.

2. Peng B, Amos CI, Kimmel M. Forward-Time Simulations of Human Populations with Complex Diseases. PLoS Genet. 2007 Mar 23;3(3):e47.

3. Jiang P-P, Corbett-Detig RB, Hartl DL, Lozovsky ER. Accessible Mutational Trajectories for the Evolution of Pyrimethamine Resistance in the Malaria Parasite Plasmodium vivax. J Mol Evol. 2013 Sep;77(3):81–91.

4. Ogbunugafor CB, Wylie CS, Diakite I, Weinreich DM, Hartl DL. Adaptive Landscape by Environment Interactions Dictate Evolutionary Dynamics in Models of Drug Resistance. PLoS Comput Biol. 2016 Jan 25;12(1):e1004710.

5. Ogbunugafor CB, Hartl DL. A pivot mutation impedes reverse evolution across an adaptive landscape for drug resistance in Plasmodium vivax. Malar J. 2016 Jan 25;15(1):40.
