# Supplementary material for: A New Take on John Maynard Smith's Concept of Protein Space for Understanding Molecular Evolution
Source: PLoS Comput Biol. 2016 Oct 13;12(10):e1005046. doi: 10.1371/journal.pcbi.1005046 (PMC5063322; doi:10.1371/journal.pcbi.1005046)
Supplement: S4 File — (DOCX) [file pcbi.1005046.s004.docx]

**Supplemental file 4**

C. Brandon Ogbunugafor and Daniel L. Hartl

***A New Take on John Maynard Smith's Concept of Protein-Space for Understanding Molecular Evolution***

**Supplementary Teaching Exercises**

Activity #1: A basic assignment featuring the updated JMS analogy

After teaching the basic premise of the device, teachers can present the following assignment that can be completed in class or for homework. It also lends itself to group work.

1. Teacher offers a **word transition schema as outlined in the manuscript (WV🡪 NY, GENE🡪BIRD etc.)**
2. **Using the Google Books *Ngram* Viewer, students construct the landscapes and graph them**
3. **Based on those results, students predict the most likely pathways that evolution will take. If there is more than one likely pathway, students should identify which are the second, third and fourth most likely pathways**
4. **After submitting the assignment, the teacher can use a simulation convention to determine the actual winners, and the probabilities that certain pathways occur relative to others**
5. **For advanced students (advanced undergraduates, graduate students and others), a teacher can require the students to find their own landscapes, graph the topography, run simulations and perhaps calculate higher-order properties of the landscapes (such as epistasis, pleiotropy and others)**
6. **Award points or prizes based on performance**

Activity #2: Classroom word evolution game

Below we outline a teaching activity, in the form of a simple game that can be used to teach principles of molecular evolution. It uses the approach as outlined in the main text. For this exercise, we’ll use a basic three-letter transition to illustrate the point how this game might play out: RAB 🡪 TIP

**Activity:**

1. Choose any relatively uncommon initial three-letter word. (Avoid initial words such as "the," "and," "for," "all," "men," "you," "our," "are," "his," etc., as these are the most commonly used three-letter words in the English language.)…. eg, RAB. By starting with a low usage first word, you’ll increase the likelihood of finding a pathway that will proceed down a pathway, as in molecular evolution.

2. Choose which letter (1, 2, 3) of this word that you will change, and change it to create a different three-letter word. Check the *n-gram* score of this word for the year 2000 (*n-gram*2000). If your new word has a larger n-gram score than the original word, keep the new word. If the *n-gram*2000 score is smaller than the original, try again until the *n-gram*2000 score has increased. (This is analogous to the change ("mutation") increasing the fitness of the organism….eg, change letter 1 R→T: RAB→TAB.)

3. From this second word, choose a different letter than you did in step 2 to change, and change it to create a different three-letter word. Check the *n-gram* score of this word for the year 2000. If your new word has a larger *n-gram* score than the word in Step 2, keep the new word. If the *n-gram*2000 score is smaller than the original, try again until the *n-gram*2000 score has increased. (This is again analogous to the change ("mutation") increasing the fitness of the organism….eg, change letter 3 B→P: RAB→TAB→TAP)

4. From this third word, choose a different letter than you did in steps 1 and 2 that you will change, and change it to create yet another three-letter word. Check the *n-gram* score of this word for the year 2000. If your new word has a larger *n-gram* score than the word in Step 3, keep the new word. If the *n-gram*2000 score is smaller than the original, try again until the *n-gram*2000 score has increased. (Another analogy to an increase in fitness of the organism….eg, change letter 2 A→I: RAB→TAB→TAP→TIP

5. Make a diagram of all six theoretical “pathways” from the first word to the last, and determine for each path whether it is possible in the sense that each word in the path has a higher “fitness” (*n-gram* score for the year 2000) than the previous word. How many of the six theoretical pathways are possible? (Greatest number of possible pathways gets a prize.)

e.g., Relative to *n-gram*2000,

1. RAB, TAB, TAP, TIP Not possible because TAP < TAB
2. RAB, TAB, TIB, TIP Not possible because TIB < TAB
3. RAB, RIB, TIB, TIP Not possible because TIB < RIB
4. RAB, RIB, RIP, TIP **Possible**
5. RAB, RAP, TAP, TIP **Possible**
6. RAB, RAP, RIP, TIP **Possible**

In this *n-gram*2000 example there are 3/6 Possible pathways from RAB to TIP

6. For the six theoretical pathways that are possible between the first word and the last, which ones are possible using as a measure of “fitness” the *n-gram* scores of the words for the year 1950. Are any pathways possible in 1950 that were no longer possible in 2000? Are any pathways not possible in 1950 that became possible in 2000? (Greatest number of changes from possible to impossible or the other way round gets a prize.)

eg, Relative to *n-gram*1950,

1. RAB, TAB, TAP, TIP **Possible** in 1950 because TAP > TAB
2. RAB, TAB, TIB, TIP Not possible in 1950 because TIB < TAB
3. RAB, RIB, TIB, TIP Not possible in 1950 because TIB < RIB
4. RAB, RIB, RIP, TIP Not possible in 1950 because RIP < RIB
5. RAB, RAP, TAP, TIP **Possible** in 1950
6. RAB, RAP, RIP, TIP **Possible** in 1950

In this *n-gram*1950 example, there are 3/6 Possible pathways from RAB to TIP, however Pathway (1), not possible in *n-gram*2000 is possible in *n-gram*1950 and Pathway (4) that is possible in *n-gram*2000 is not possible in *n-gram*1950. In this case the score of possible to impossible or the other way round equals 2.

**From this basic exercise, one might introduce several game variants:**

1) Assign the same initial word to all students or groups and have them evolve as above. Do any end up with the same final word? Did they get there by the same pathway? This would be an example of *parallel evolution*. There could be a prize for the highest scoring final word.

2) Assign different initial words to all students or groups and have them evolve as above. Do any end up with the same final word? Did they get there by the same pathway? This would be an example of *convergent evolution*.
