# Supplementary material for: A New Take on John Maynard Smith's Concept of Protein Space for Understanding Molecular Evolution
Source: PLoS Comput Biol. 2016 Oct 13;12(10):e1005046. doi: 10.1371/journal.pcbi.1005046 (PMC5063322; doi:10.1371/journal.pcbi.1005046)
Supplement: S5 File — (PDF) [file pcbi.1005046.s005.pdf]

A New Take on John Maynard Smith's Concept of Protein Space for Understanding Molecular Evolution

An educator's guide to a new model for exploring advanced topics in molecular evolution

C. Brandon Ogbunu and Daniel L. Hartl
Department of Organismic and Evolutionary Biology, Harvard University

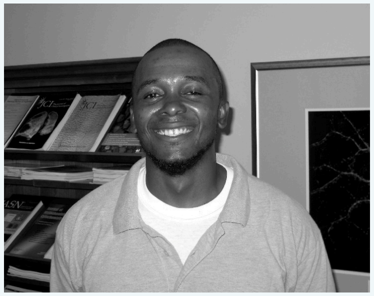

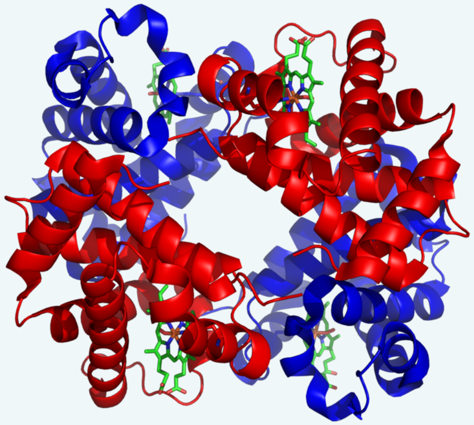

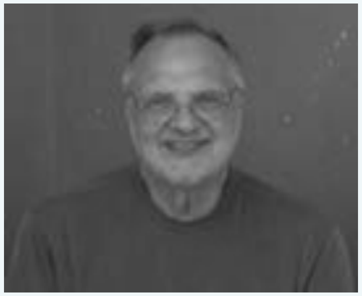

Basic questions

- What is the modern state of evolutionary understanding?
  - How/is the number of people who understand Darwinian evolution changing?
  - Is evolutionary understanding improving in *quality*?
- What are the barriers to a better grasp of evolutionary biology?
- Can we create new ways to teach and communicate these ideas?

Barriers to progress in teaching and understanding evolutionary biology

- The problem of scale
  - Requires one to comprehend objects (organisms, mutations and time) in larger numbers and on longer time scale than any normal cognitive task
- Statistical reasoning
- Population-level thinking
- Incremental nature of evolution
- More detailed understanding requires a generally quantitative toolkit

Metaphors and analogies in science

- **Especially useful for abstract or non-intuitive ideas**
- Michelson-Morley experiment (physics)
- Schrodinger's cat (quantum mechanics)
- The Selfish Gene[a], The Blind Watchmaker[b], etc. The fitness landscape (evolutionary biology)
- **The "change-one-letter" game as a metaphor for evolution in protein space** (evolutionary biology)

[a]Dawkins, Richard (1976). *The Selfish Gene*. Oxford. Oxford University Press.
[b]Dawkins, Richard (1986). *The Blind Watchmaker.* New York. W.W. Norton & Company, Inc.
See main text for references to the other examples

Protein evolution

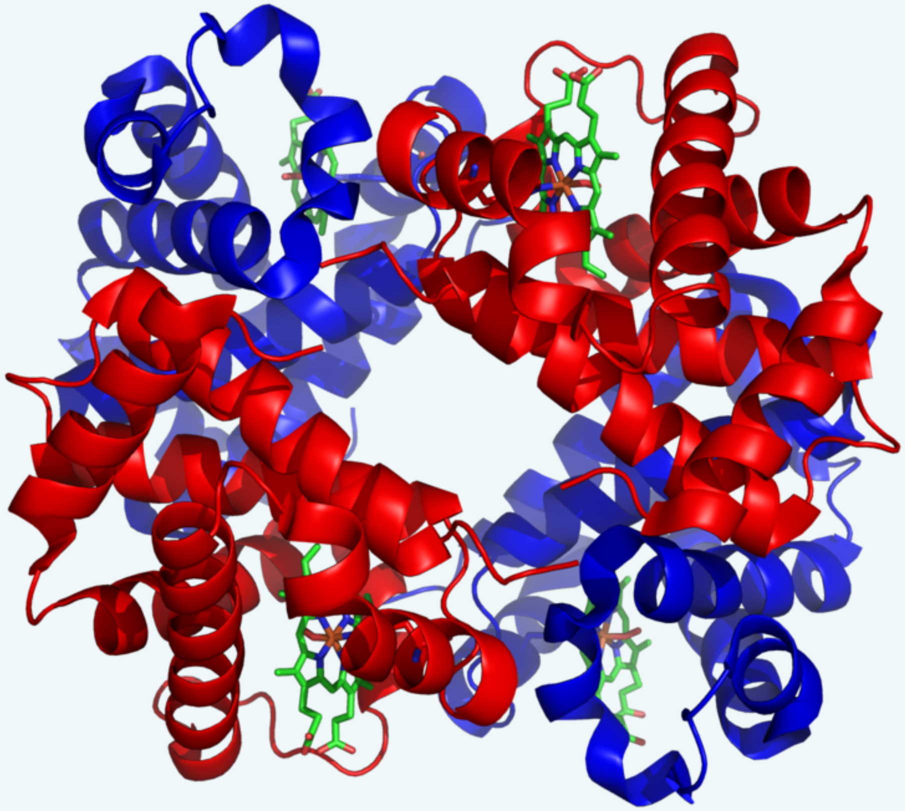

https://en.wikipedia.org/wiki/Hemoglobin#/media/File:1GZX_Haemoglobin.png

Protein evolution: The change-one-letter game
(e.g., WORD→→→→GENE)

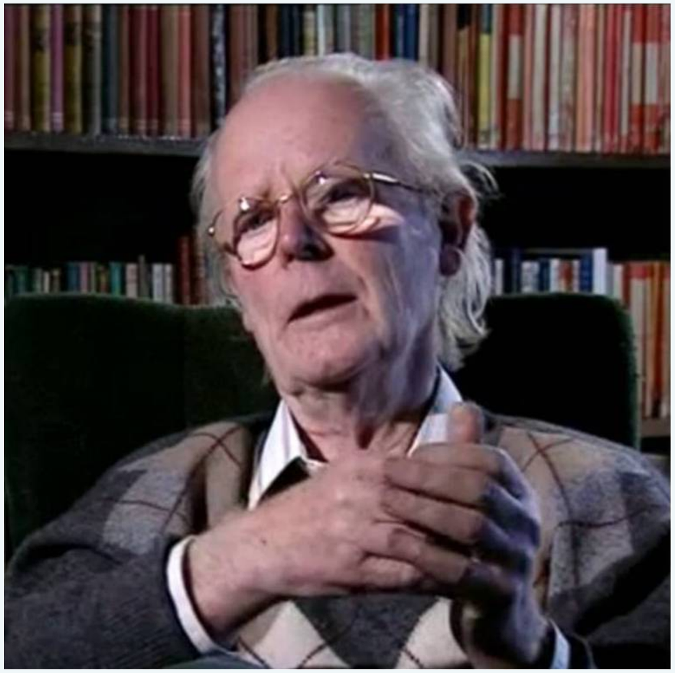

John Maynard Smith

Photo source: https://en.wikipedia.org/wiki/John_Maynard_Smith#/media/File:John_Maynard_Smith.jpg

WORD→→→→GENE

Which of these pathways is the "best"?

1. WORD→GORD→GORE→GONE→GENE
2. WORD→WERD→WERE→WENE→GENE
3. WORD→WERD→GERD→GERE→GENE
4. WORD→WORE→GORE→GONE→GENE
5. WORD→WOND→WONE→GONE→GENE

Maynard Smith, J. *Nature* **225**, 563 - 564 (07 February 1970)

WORD→→→→GENE

Which of these pathways is the "best"?

1. WORD→GORD→GORE→GONE→GENE
2. WORD→WERD→WERE→WENE→GENE
3. WORD→WERD→GERD→GERE→GENE
4. WORD→WORE→GORE→GONE→GENE
5. WORD→WOND→WONE→GONE→GENE

The one (4) where each of the individual words makes sense along the way! This is how protein space is constructed: functional proteins exist in networks, and are located through mutation and selection

Maynard Smith, J. *Nature* **225**, 563 - 564 (07 February 1970)

WORD→→→→GENE

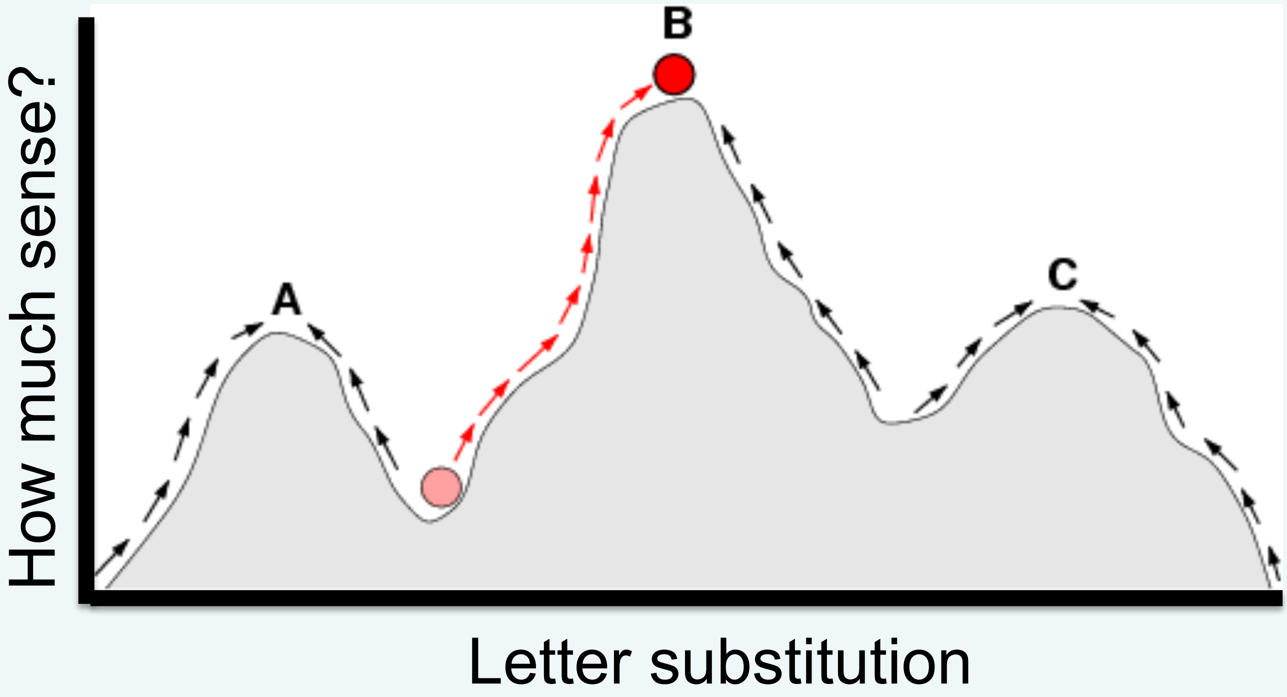

One can easily transform the Maynard Smith analogy into an **adaptive landscape**. To do so, each of the words in Maynard Smith's protein space analogy need values associated with how much "sense" it makes, a proxy for its biological "fitness."

Image source: https://en.wikipedia.org/wiki/Fitness_landscape#/media/File:Fitness-landscape-cartoon.png

The adaptive landscape analogy in action: The Weinreich-Hartl Method (2006)*

- By understanding the individual fitness of mutant alleles, you can determine the relative accessibility of pathways towards maximal drug resistance
- This landmark approach has been especially useful in studying the evolution of drug resistance

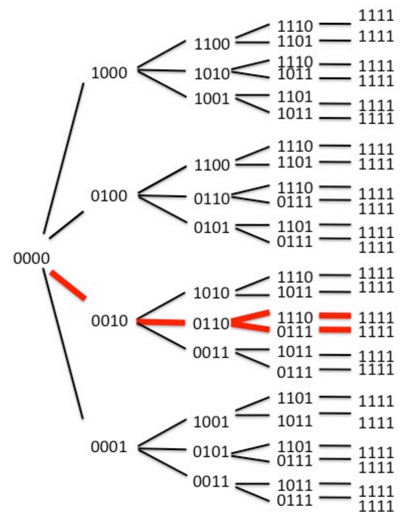

See main text of manuscript for more details

*Weinreich DM, Delaney NF, Depristo MA, Hartl DL. Darwinian evolution can follow only very few mutational paths to fitter proteins. Science. 2006 Apr 7;312(5770):111–4.

Lozovsky ER, Chookajorn T, Brown KM, Imwong M, Shaw PJ, Kamchonwongpaisan S, et al. Stepwise acquisition of pyrimethamine resistance in the malaria parasite. Proc Natl Acad Sci U S A. 2009 Jul 21;106(29):12025–30

What types of big questions can be addressed?

- How does natural selection work?
- If the forces that generate variation are random, how can natural selection "find" adaptive solutions?
- To what extent is evolution repeatable? Are some evolutionary pathways more likely than others?

Objectives

1. Reimagine the **WORD→→→→GENE** metaphor towards a sort of game to understand molecular evolution
2. Use a reimagined adaptive landscapes to explore both classical and modern concepts in evolutionary genetics
3. Propose methods for using this reimagined landscape as a pedagogical tool

Adaptive landscape: Google *n-gram* (1)

- Original WORD →→→→GENE analogy was entirely arbitrary, because the nodes in the "fitness landscape" lacked any fitness correlate
- Computational linguistics
- How does it work?
- Use Google *n-gram* scores as fitness proxies in the WORD→→→→GENE landscape

Adaptive landscape: Google *n-gram* (2)

- Fitness parameters for a single landscape with multiple environmental dimensionality: time -- anything between 1800-2000 (and sometimes earlier) and language (over a dozen)

- With this tool, one can transform an abstract metaphor for evolution in protein sequence space into a more concrete and game-like pedagogical tool

Methods

1. Identify transitions
2. Google *n-gram* fitness values for all possible alleles in the adaptive landscape
3. Reconstruct pathways
4. Identify most likely pathways
5. Simulations to verify most likely pathways

Concepts to explore

- The concept of protein space
- The incremental nature of evolution
- The adaptive landscape
  – Evolutionary pathway (trajectory)
- Gene × environment interactions (advanced students)
- Epistasis (advanced students)
- Evolutionary simulations (advanced)

Learning path: Beginner

- Citizen-science courses for improving the general public's understanding of science
- High school-level biology
- College: introductory biology, biology courses for non-majors
- Concepts:
  - The central dogma of biology
  - The concept of mutation
  - The basics of Darwinian evolution

Learning path: Intermediate

- High school: advanced students only
- College: biology, genetics and evolution
- Concepts:
  - The concept of protein space, as described by John Maynard Smith
  - Gene by environment interactions
  - The fitness/adaptive landscape
- Can be integrated with courses in statistics and computer science

Learning path: Advanced

- College: upper-division undergraduate courses in evolution, genetics and computational biology
- Introductory graduate seminars
- Concepts:
  - Epistasis
  - Pleiotropy and tradeoffs
  - Fitness landscape topography
  - Evolvability
- Can be integrated with courses in statistics and computer science

For advanced students: Epistasis

- Measure the average effect of a mutation as a function of genetic background
- Measure the interaction between epistasis and environment

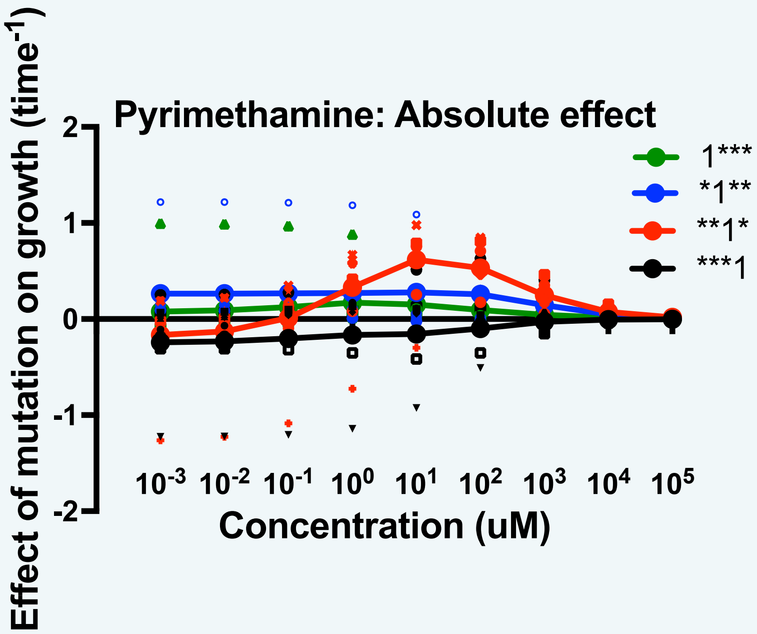

Ogbunugafor CB et al. Adaptive Landscape by Environment Interactions Dictate Evolutionary Dynamics in Models of Drug Resistance. PLOS Comput Biol. 2016 Jan 25;12(1):e1004710

Proposed exercise for beginning and intermediate level learners

- Teacher offers a word transition schema as outlined in the manuscript (e.g., WV→→NY, RAB→→→TIP, GENE→→→→BIRD)*
- Using the Google Books *Ngram* Viewer, students construct the landscape
- Students predict the most likely trajectories
- Teacher can use computer simulations of "mutation" to determine the actual winners, and the probabilities that certain pathways occur relative to others
- Award points or prizes based on performance

*See **Supplementary File 4** for more details

Proposed exercise for advanced learners

- Similar to the beginning/advanced exercise, but using more complicated landscapes with more nodes (letters)
- For very advanced students, require the learners to identify their own word-transition landscapes, graph the topography, run simulations and calculate higher-order properties of the landscape
- Calculate other properties: G × E effects and epistasis

Evaluation

- Track their performance across the actual exercise (how well they perform in identifying and creating word landscapes)
- Test general mastery of evolutionary principles
  - Do they grasp the basic tenets of evolutionary biology?
  - Ask students to define evolution
  - Answers to questions: how does evolution find biological solutions to problems amidst a large number of possibilities?
- Teach the seminal study: Weinreich et al. (2006)*
  - Now that students been presented with a new perspective on adaptive trajectories via the word-evolution analogy, does the manuscript make more sense?
  - Gauge student performance on examinations

*Weinreich DM, Delaney NF, Depristo MA, Hartl DL. Darwinian evolution can follow only very few mutational paths to fitter proteins. Science. 2006 Apr 7;312(5770):111–4.

Conclusions I

- General properties of evolutionary genetics playing out in a metaphorical, easy to understand, and gamelike space
- Can explore cutting-edge ideas in genetics, evolutionary biology, and network theory (e.g., gene by environment interactions, adaptive landscape by environment interactions, robustness, evolvability)

Conclusions II

- We’ve created a new pedagogical tool to help drive home the elusive incremental nature of evolutionary change

- Additional benefits: can be integrated with coursework in mathematics, computer science, or statistics

Future

- Capitalize on *multiplicity*: Any number of word transitions can be used to create an Vast number of landscapes
- Align with curricula in biology (and other courses)

Contact

C. Brandon Ogbunu
scholar.harvard.edu/chike98
ogbunugafor@oeb.harvard.edu
Twitter: @Word2Gene
