# Supplementary material for: A New Take on John Maynard Smith's Concept of Protein Space for Understanding Molecular Evolution
Source: PLoS Comput Biol. 2016 Oct 13;12(10):e1005046. doi: 10.1371/journal.pcbi.1005046 (PMC5063322; doi:10.1371/journal.pcbi.1005046)
Supplement: S6 File — (DOCX) [file pcbi.1005046.s006.docx]

**Supplemental file 6**

C. Brandon Ogbunugafor and Daniel L. Hartl

***A New Take on John Maynard Smith's Concept of Protein-Space for Understanding Molecular Evolution***

**Additional references on adaptive landscapes**

For those looking for broad reviews on the topic of empirical adaptive landscapes, we highlight two good summaries:

de Visser JAGM, Krug J. Empirical fitness landscapes and the predictability of evolution. Nat Rev Genet. 2014 Jul;15(7):480–90.

Poelwijk FJ, Kiviet DJ, Weinreich DM, Tans SJ. Empirical fitness landscapes reveal accessible evolutionary paths. Nature. 2007 Jan 25;445(7126):383–6.

Additionally, below we provide a set of references for those interested in further exploring the study of adaptive landscapes in greater detail. This is by no means an exhaustive list, but only a small sample of the types of work being conducted across a number of systems.

Aita T, Iwakura M, Husimi Y. A cross-section of the fitness landscape of dihydrofolate reductase. Protein Eng. 2001 Sep 1;14(9):633–8.

Hietpas RT, Jensen JD, Bolon DNA. Experimental illumination of a fitness landscape.
Proc Natl Acad Sci. 2011 May 10;108(19):7896–901.

Inoguchi N, Weber RE, Fago A, Moriyama H, Storz JF. Epistasis Among Adaptive Mutations in Deer Mouse Hemoglobin. Science. 2013 Jun 14;340(6138):1324–7.

Jiménez JI, Xulvi-Brunet R, Campbell GW, Turk-MacLeod R, Chen IA. Comprehensive

experimental fitness landscape and evolutionary network for small RNA. Proc Natl

Acad Sci. 2013 Sep 10;110(37):14984–9.

Kouyos RD, Leventhal GE, Hinkley T, Haddad M, Whitcomb JM, Petropoulos CJ, et al. Exploring the complexity of the HIV-1 fitness landscape. PLoS Genet. 2012;8(3):e1002551.

Lauring AS, Andino R. Exploring the fitness landscape of an RNA virus by using a universal barcode microarray. J Virol. 2011 Apr;85(8):3780–91.

Leventhal GE, Hinkley T, Haddad M, Whitcomb JM, Petropoulos CJ, Bonhoeffer S.

Exploring the Complexity of the HIV-1 Fitness Landscape. PLoS Genet. 2012 Mar

8;8(3):e1002551.

Lindsey HA, Gallie J, Taylor S, Kerr B. Evolutionary rescue from extinction is contingent on a lower rate of environmental change. Nature. 2013 Feb 28;494(7438):463–7.

Martin CH, Wainwright PC. Multiple Fitness Peaks on the Adaptive Landscape Drive

Adaptive Radiation in the Wild. Science. 2013 Jan 11;339(6116):208–11.

Nahum JR, Godfrey-Smith P, Harding BN, Marcus JH, Carlson-Stevermer J, Kerr B. A tortoise–hare pattern seen in adapting structured and unstructured populations suggests a rugged fitness landscape in bacteria. Proc Natl Acad Sci. 2015 Jun 16;112(24):7530–5.

Ostrowski EA, Ofria C, Lenski RE. Genetically integrated traits and rugged adaptive landscapes in digital organisms. BMC Evol Biol. 2015;15:83.

Palmer AC, Toprak E, Baym M, Kim S, Veres A, Bershtein S, et al. Delayed commitment to evolutionary fate in antibiotic resistance fitness landscapes. Nat Commun. 2015 Jun 10;6:7385.

Payne JL, Wagner A. The robustness and evolvability of transcription factor binding sites. Science. 2014 Feb 21;343(6173):875–7.

Rozen DE, Habets MGJL, Handel A, Visser JAGM de. Heterogeneous Adaptive Trajectories of Small Populations on Complex Fitness Landscapes. PLOS ONE. 2008 Mar 5;3(3):e1715.

Sarkisyan KS, Bolotin DA, Meer MV, Usmanova DR, Mishin AS, Sharonov GV, et al. Local fitness landscape of the green fluorescent protein. Nature. 2016 May 11
