# Supplementary material for: A New Take on John Maynard Smith's Concept of Protein Space for Understanding Molecular Evolution
Source: PLoS Comput Biol. 2016 Oct 13;12(10):e1005046. doi: 10.1371/journal.pcbi.1005046 (PMC5063322; doi:10.1371/journal.pcbi.1005046)
Supplement: S7 File — (DOCX) [file pcbi.1005046.s007.docx]

**Supplemental file 7**

C. Brandon Ogbunugafor and Daniel L. Hartl

***A New Take on John Maynard Smith's Concept of Protein-Space for Understanding Molecular Evolution***

**Two letter U.S. state abbreviation 1-grams example**

To further illustrate how the Google Books *Ngram* Viewer can be used to rewire the John Maynard Smith analogy into a way to explore the adaptive landscape concept, we can use a simple example: transitions between two-letter 1-grams involving abbreviations for U.S. States. We’ll use the transition from **WV**, the abbreviation for the state of West Virginia, into **NY**, the abbreviation for the state of New York.

**WV🡪NY**

We can also represent these *n-grams* in bit string notation:

**00🡪11**

Where **WV** = **00** and **NY** = **11**.

In this scenario, there will be N! pathways between 00 and 11, or 2 for a two letter 1-gram (2!). For this transition, we can represent all “pathways” between the **WV** and **NY** with:

**00🡪01🡪11 = WV🡪WY🡪NY**

**00🡪10🡪11 = WV🡪NV🡪NY**

For this example, both pathways appear accessible, as the intermediates (**WY** and **NV**) correspond to actual state abbreviations (Wyoming and Nevada, respectively). The problem is that the original JMS analogy didn’t intend to discuss the concept of the adaptive landscape, and so offered no means of differentiating between the likelihood of these two pathways.

Using the Google Books *Ngram* Viewer described above (and in Box 1), we can create a table of *n-gram* fitness values (*n-gram* frequencies) for the individual two-letter 1-grams from 1800 to 2000 (Table S1). From this, we can reconstruct the *n-grams* into discrete pathways (S1 Fig.). A pathway is accessible if each step consisted of a word (1-gram) that was used more frequently than the previous word in the path. We can observe that in the year 2000, both pathways between **WV🡪NY** are feasible, but to a slightly different degree: In 2000, **NV** (Nevada) has higher *n-gram* fitness than Wyoming (**WY**), and is therefore a higher fitness intermediate, making its pathway (**WV🡪NV🡪NY**) more likely. Note, however, that in our data set, the 1800 and 1900 trajectories present a different scenario: neither **NV** nor **WY** have a higher fitness than **WV**, suggesting that routes to **NY** are unlikely in either of these environments. This is another illustration of a key point: how gene by environment interactions (G x E) create changes in adaptive landscape structure and alter the feasibility of certain trajectories (as discussed in the main text).

**
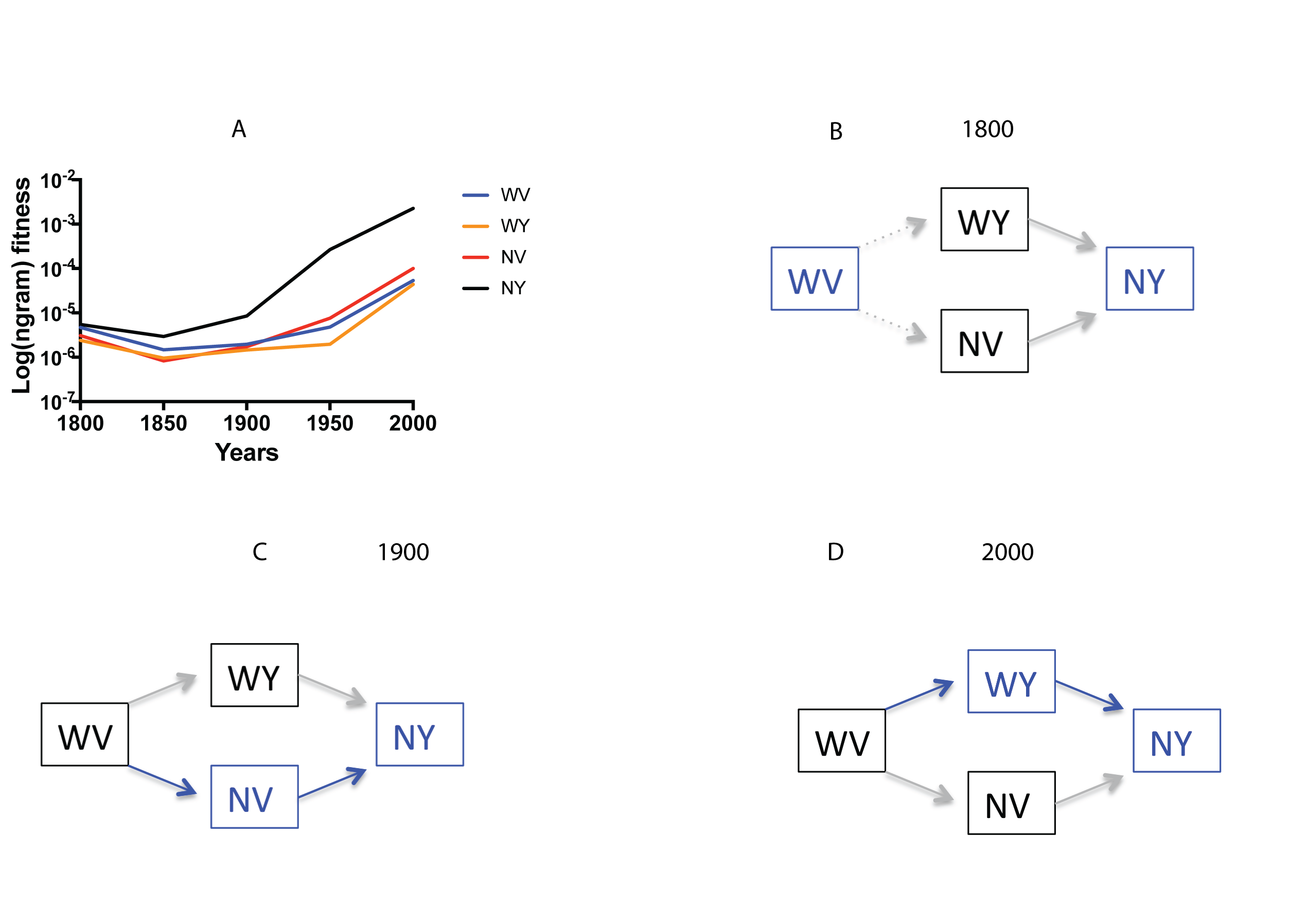
**

**S7A-D Fig. Creating a model two-letter *1-gram* adaptive trajectory: WV🡪NY.** (A) *Ngram* Fitness values for the alleles composing the landscape across environments (year), plotted on a log scale (*y*-axis). From the information in (A), we can identify probable pathways between 1-grams in (B) 1800, (C) 1900 and (D) 2000. In this example, we observe that the transition between **WV🡪NY** is unlikely at 1800 and 1900 because **WV** has higher *n-gram* fitness than either intermediate (**WY** **or NV**). By 2000 this has changed, with **NV** having high *n-gram* fitness than the starting “allele,” **WV** (and higher than that of **WY)**, revealing an accessible pathway through sequence space between **WV** and **NY: WV🡪NV🡪NY.** Note that in 2000, **WV🡪WY🡪NY** is also accessible by our criteria (increasing fitness with Hamming distance from **WV** to **NY**). However, because **NV** has higher *n-gram* fitness than **WY**, the more probable pathway is **WV🡪NV🡪NY**. In Supplemental File 3, we discuss how to examine this scenario using simulations of evolution.

| **State Abbreviation** | **1-gram frequency score**  **(Year 1800)** | **1-gram frequency core**  **(Year 1850)** | **1-gram frequency score**  **(Year 1900)** | **1-gram frequency score**  **(Year 1950)** | **1-gram frequency score**  **(Year 2000)** |
| --- | --- | --- | --- | --- | --- |
| **WV** | 4.69E-06 | 1.48E-06 | 1.97E-06 | 4.81E-06 | 5.38E-05 |
| **WY** | 2.40E-06 | 9.58E-07 | 1.46E-06 | 1.96E-06 | 4.39E-05 |
| **NV** | 3.10E-06 | 8.29E-07 | 1.73E-06 | 7.64E-06 | 1.01E-04 |
| **NY** | 5.49E-06 | 2.94E-06 | 8.57 E-06 | 2.68E-04 | 2.27E-03 |

**S7 Table.** *N-gram* frequency scores (*Ngram* fitness) for several U.S. state abbreviations (WV, WY, NV, NY) for five different years from (1800, 1850, 1900, 1950, 2000). Note that these frequency scores apply to the case sensitive versions of the words, because state abbreviations, written correctly, are always uppercase.


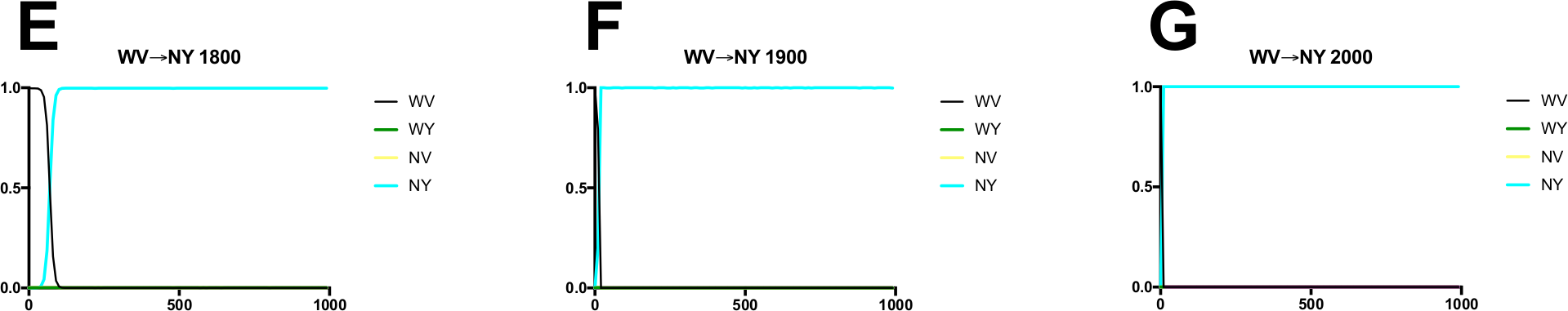


**S7E-G Figs. Simulations of evolution across the WV🡪NY landscape.** These are illustrative examples of the results of simulations of evolution from **WV** to **NY** across several environments: (A) 1800, (B) 1900 (C) 2000, using simuPOP, as mentioned in Supplemental File 3. The mutation rate in this experiment is on the order of 1 mutation per replication, and they were run for 1000 generations (100,000 individuals). We can observe that leaving the WV peak is slightly more difficult to do in 1800 than in 1900 or 2000, as it takes slightly longer for the NY “allele” to take over the population.
